# Critical review on chemical compositions and health-promoting effects of mushroom *Agaricus blazei* Murill

**DOI:** 10.1016/j.crfs.2022.10.029

**Published:** 2022-11-05

**Authors:** Kaiyuan Huang, Hesham R. El-Seedi, Baojun Xu

**Affiliations:** a Food Science and Technology Program, Department of Life Sciences, BNU-HKBU United International College, Zhuhai, China; b Department of Chemistry, The Hong Kong University of Science and Technology, Hong Kong, China; c Pharmacognosy Group, Department of Pharmaceutical Biosciences, Uppsala University, Biomedical Centre, Box 591, SE, 751 24, Uppsala, Sweden; d International Research Center for Food Nutrition and Safety, Jiangsu University, Zhenjiang, 212013, China

**Keywords:** *Agaricus blazei Murill*, Phytochemical constituents, Immunoregulation, Inflammation, Pharmacological uses

## Abstract

*Agaricus blazei* Murrill (AbM) is a medical mushroom which has huge potential commercial value with various health-promoting functions. However, the chemical composition and therapeutic mechanisms of AbM have not been concluded systematically yet. Thus, this study aims to comprehensively summarize the phytochemical profiles and thoroughly characterize the health promotion effects such as the antitumor and antidiabetic impact of AbM in *in vivo* and *in vitro*. The AbM consists of abundant bioactive substances; polysaccharides, lipids including ergosterol, sterols, proteins, vitamin B, C and D, and phenolic compounds. Several studies have claimed that *Agaricus blazei* Murrill polysaccharides (AbMP) had immunoregulation, anti-inflammatory, hepatoprotective, and antitumor function both *in vivo* and *in vitro*. Meanwhile, AbM extracts were thought to cure diabetes and bacterial infection, exhibiting anticarcinogenic and antimutagenic functions. But some principles behind health-promoting effects have not been clarified. Additionally, AbM related clinical trials are limited and further discovery need to be conducted. Therefore, this paper has concluded the health promotion impact with corresponding mechanisms of AbM and indicated its potential medical usage as functional food in the future.

## Highlights

• Health-promoting functions of AbM and potential mechanisms were comprehensively summarized.
• Mechanism model of inflammation inhibitory effects of AbM was described.
• Impact of different food processing on antioxidants, nutrients is fully discussed.

## Graphical abstract

**Figure:**
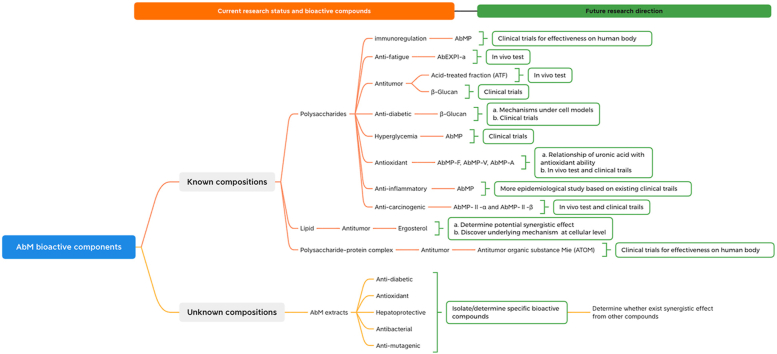

## 1 Introduction

Mushrooms have been considered a functional and nutritional food worldwide due to their low calories and high content of bioactive components, proteins, lipids, minerals, and vitamins. In the early 21st century, higher basidiomycetes as medical mushrooms have attracted much attention due to their nutritional and pharmacological properties (Delmanto et al., 2001). *Agaricus blazei* Murill (AbM), scientific name Cyperus mushroom, is an edible *Basidiomycetes* mushroom, which was first discovered in Florida, U.S.A., in 1944 and is known as Himematsutake in Japan and Ji-songrong in China (Wu and Wang, 2000). AbM was first brought to Japan in 1965; with the maturity of artificial cultivation technology, it has been widely used in tea and food, and later gained a reputation for its excellent medical value and biochemical properties since the 1980s. In many countries, such as in Brazil, people use the extracts as natural therapy and tea, which is widely accepted as a new method to prevent and treat cancer (Delmanto et al., 2001; Mizuno, 2002).

**Fig. 1:**
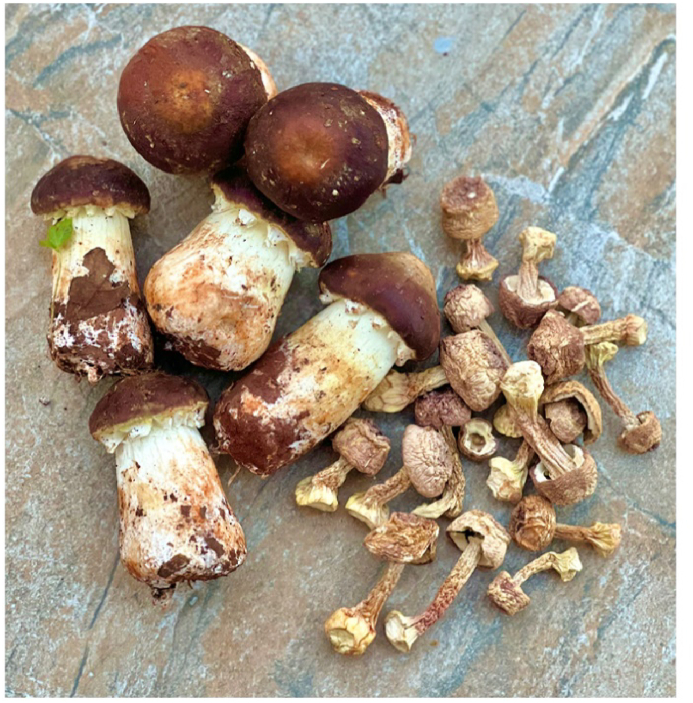
Agaricus blazei Murill (AbM) fresh and dry samples were purchased in Taobao on June 29, 2022 in Yunnan province, China.

Murrill (1945) comprehensively described the morphological characteristics of AbM; the cap of was initially hemispherical, up to 1–1.5 cm thick in the centre, 7–9 cm broad, margin glabrous, flat in the middle, and its color ranged from white to light gray or dark reddish-brown. Then it becomes convex with a diameter of 6–11 cm, and the surface was covered with filamentous fibers which would form small scales when matured. The spores exhibited dark brown color, smooth, broadly oval, and spherical, about 5 × 4 μm, without bud holes, and the hyphae had no lock-like association (Figure 1). Tian and Ren (2009) further claimed that AbM cultivation would be beneficial to the development of circular agriculture and conservation agriculture. As a saprophytic fungus, AbM can be planted entirely with crop straw, while operations such as bagging and sterilization are not in need. On the other hand, picking process of AbM is convenient with a high input-output ratio since it be refrigerated for freshness or dried and dehydrated for transportation or storage. Moreover, the optimum temperature ranged from 22 to 26°C, and 60–70% moisture content without light and a 6–7.5 pH value should be maintained to the culture material.

In China, the concept of homology of medicine and food occupies an important position in traditional Chinese medicine theory (Zhou and Xu, 2021). On the other hand, AbM had a relatively short history of consumption in China, which was first introduced to Fujian Province in China from Japan in 1992. Since then, Chinese scientists are more enthusiastic on the research of health promotion function of the mushroom in the past few decades. AbM has been evidenced by several *in vivo* and *in vitro* studies since 21st and utilized medicinally to treat various diseases including diabetes, arteriosclerosis, chronic hepatitis, hyperlipidemia, and cancer (Hetland et al., 2008). Additionally, AbM was capable of regulating cellular immunity, antioxidant, antibacterial and anti-inflammatory responses (Firenzuoli et al., 2008). Wang et al. (2014) and Halpern (2007) proved that AbM could significantly strengthen the immune system by improving the activities of natural killer cells and increasing the number of white blood cells. And it was also generally believed that AbM extracts exhibited antioxidant, hepatoprotective and anti-viral activities (Sorimachi et al., 2001). Thus, nutrition compositions of AbM aroused heated attention among researchers.

Ohno et al. (2001) systematically investigated the constituents of AbM and found it mainly consisted of polysaccharides, (1–3)-β-D-β-glucans with side branches of a (1–6)-β-backbone, generating considerable immunomodulatory and antitumor effects by activation of cytotoxic macrophages, helper T cells, and NK cells, promotion of T cell differentiation, and activation of the alternative complement pathway. The authors suggested high molecular weight of triple helical structure may be responsible for immunopotentiation activity, but few data can indicate was independent of any specific ordered structure. More recently, Menezes et al. (2022) demonstrated that AbM polysaccharides (AbMP) had good rheological properties, and which can be used to develop pharmaceutical formulations with antioxidant activity and low cytotoxicity to human neutrophils. Furthermore, sulfated-modified AbMP were claimed to increase their anti-HIV pharmacological activity, holding the promise for a future biomedical treatment of HIV/AIDS (Zhao et al., 2021). Besides, ergosterol, firstly isolated by Takaku et al. (2001) from AbM lipid was demonstrated its significant tumor growth inhibition ability *in vivo* study without side effects. In addition, vitamin B_1_, B_2_, B_9,_ B_12_, vitamin C, vitamin D and niacin could also be found in the AbM fruit body (Mizuno, 1995; Rózsa et al., 2019). The scientists suggested that these pharmacological nutrients can be isolated from different parts of the mushroom. In particular, fruit bodies, pure culture mycelia, and culture filtrates were considered fundamental parts that were rich in bioactive nutrients extracted by microwaving, organic solvents or hot water extraction and centrifuging (Sun et al., 2011; Shen et al., 2001; Li, 2020). An increasing number of scientists are trying to obtain active metabolites from mycelia through deep fermentation to obtain cheaper preparations for immunoregulation or disease treatment (Firenzuoli et al., 2008). Some compositions may not isolate from the mushroom; however, they still expressed the bioactive functions overall. Sorimachi et al. (2001) reported that AbM mycelium also revealed cytopathic inhibition effects of western equine encephalitis virus on cultured VERO cells. Fan et al. (2006) documented the efficacy of the AbM compound combined with chemotherapy for gastric cancer. They confirmed that white cells and CD4^+^/CD8^+^ significantly increased after treatment, which indicated that AbM could relieve clinical symptoms with the combination of chemotherapy. Beyond that, some AbM-based medical products have been developed and evidenced by clinical trials. AndoSan™ was one of the clinical anti-inflammatory medicines which can treat Crohn's disease (CD) by reducing pro-inflammatory cytokines in patients (Therkelsen et al., 2016). Although existing clinical studies on the pharmacological effects of AbM were relatively limited, it has been widely used and considered complementary and alternative medicine in worldwide (Wang et al., 2013).

Existing reviews have rarely concluded for their nutrition composition and potential therapeutic effects of AbM. Thus, this critical review aims to comprehensively summarize AbM chemical profiles with their nutritional value; the existing bioactivities of AbM. On the other hand, some mechanisms were thoroughly summarized for the further clinical application and bioactive principal discovery. Also, the impact of food processing of AbM were compared. The review collected the previous studies across the past 40 years via screening of databases such as PubMed, Google Scholar, ResearchGate, etc., using the corresponding keywords. The tables and figures were generated by Excel (version 16.62), XMind and BioRender.

## 2 Nutritional values of AbM

AbM as a functional food, which enjoyed great popularity for numerous high-quality bioactive nutrient components. In general, active metabolites can be isolated from AbM fruiting bodies, pure cultured mycelium, submerged fermentation culture from mycelium and culture filtrate. There are differences in the content of biologically active substances in different parts of AbM (mycelia and fruit body) and between treatment methods (dry powder, hydroalcoholic extracts and fresh AbM). The detail gross compositions of mushrooms and the contents of different parts were collected in Table 1. The AbM comprised vibrant carbohydrates (59.42 g/100g), such as oligosaccharides, mannans, xylose, *α*-glucans, and *β*-glucans and mainly contained four monosaccharides: glucose, galactose, mannose and fucose (Carneiro et al., 2013; Kim et al., 2005; Liu et al. 2022; OHNO et al., 2001; Mizuno et al., 1990). Moreover, AbMP including AbEXP1-a (Li, 2020), AbMP-Ⅱ-α and AbMP-Ⅱ-β (Shen et al., 2001), FI_0_-a-β, FA-1-a-⍺, FA-a-β (Mizuno et al., 1990), agaritine (Akiyama et al., 2011) could be extracted from AbM. Uronic acid as an acid glucan accounted for about 28.19%, which may potentially affect their bioactive activities (Wu et al., 2014). On the other hand, ergosterol (73–90 mg/100 g) was isolated from AbM lipid fractions by Takaku et al. (2001). Ergosterol, as a precursor of vitamin D_2_ (1.95–3.68 mg/100g), was usually present in AbM mushrooms at higher levels than other crops (Rózsa et al., 2019; Vieira Junior et al., 2021). Normally, fat accounted for 1.82 g/100 g (2–8%) of the AbM (Carneiro et al., 2013; Firenzuoli et al., 2008). In addition, fiber (3–32%) and vitamin C (11–21 mg/100g) were rich in common edible mushrooms including AbM (Horm and Ohga 2008; Takaku et al., 2001). Vitamin B_1_, B_2_, B_9,_ B_12_ and niacin could be found in AbM, especially in fruit organisms (Mizuno, 1995; Rózsa et al., 2019). Moreover, Sun (2007) claimed that the mycelium and fruiting body of AbM contain various essential trace elements, especially zinc (Zn) and selenium (Se). Hemagglutinin (ABL) as a lectin that can be extracted from the fruiting body of fresh AbM (Kawagishi et al. 2001).

**Table 1:** The relative proportion of nutritional compounds and phytochemicals in *Agaricus blazei Murill (AbM)*.

Composition	Content (mean ± SD, percentage, or content range)	Reference
Carbohydrates	59.42 ± 1.86 g/100g (DP); 121.2 ± 9.20 μg/mg (FE)*	Carneiro et al. (2013); Carvajal et al., 2011
Fat	1.82 ± 0.03 g/100 g (DP); 2–8 % (FW)	Carneiro et al. (2013); Firenzuoli et al. (2008)
Proteins	31.29 ± 1.85 g/100g (DP); 2–40 % (FW)	Carneiro et al. (2013); Firenzuoli et al. (2008)
Total sugars	66.91 ± 7.58 g/100 g (DP)	Carneiro et al. (2013)
Total soluble sugar (g/100g)	1.72 ± 0.17 (FW)	Sun et al. (2011);
Crude Ash	5–7 %; 7.47 ± 0.04 (DP)	Takaku et al. (2001); Carneiro et al. (2013)
Fiber	3–32 % (FW)	Takaku et al. (2001); Firenzuoli et al. (2008)
Moisture	85–90 % (FW)	Takaku et al. (2001); Firenzuoli et al. (2008)
Total tocopherols	124.25 ± 31.30 μg/100g (DP)	Carneiro et al. (2013)
Total phenolic and related compounds	0.77 ± 0.12 mg/100g (DP)	Rózsa et al. (2019)
Uronic acid	28.19 ± 1.39% (Freeze drying)	Wu et al. (2014)
Mannose	49.12–80.71 mg/100g (FW); 0.59 % (DP)	Sun et al. (2011); Liu et al. (2017)
Trehalose	23.9 ± 0.24 mg/g (MY); 5.74 ± 0.70 mg/100g (DP)	Chang et al. (2001); Carneiro et al. (2013)
Arabitol	31.4 ± 0.68 mg/g (MY)	Chang et al. (2001)
Galactose	5.46 % (DP); 210.43 ± 8.22 mg/100g (FW)	Liu et al. (2017); Sun et al. (2011)
Glucose	403.19–852.23 mg/100 g (FW); 83.59 % (DP)	Sun et al. (2011); Liu et al. (2017)
Ergosterol	73.00–90.17 mg/100g (DP)	Rózsa et al. (2019)
Mannitol	7.94 mg/100g (DP)	Carneiro et al. (2013)
Flavonoids	1.8 ± 0.16 μg/mg (FE)*	Carvajal et al. (2011)
Fructose	0.27 ± 0.02 g/100 g (DP); 1.04 % (DP)	Carneiro et al. (2013); Liu et al. (2017)
L-Aspartic acid	0.50 ± 0.06 mg/g (MY)	Chang et al. (2001)
L-Glutamic acid	nd (MY)	Chang et al. (2001)
*p*-hydroxybenzoic acid	0.64 ± 0.09 mg/100g (DP)	Carneiro et al. (2013)
Vanillic acid	nd (DP)	Carneiro et al. (2013)
Trans-*p*-coumaric acid	0.08 ± 0.02 mg/100g (DP)	Carneiro et al. (2013)
Gallic acid	4.50 ± 0.10 μg/mg (FE) *	Carvajal et al. (2011)
Syringic acid	5.70 ± 0.10 μg/mg (FE) *	Carvajal et al. (2011)
Pyrogallo acid	3.5 ± 0.10 μg/mg (FE) *	Carvajal et al. (2011)
Cinnamic acid	0.05 ± 0.01 mg/100g (DP)	Carneiro et al. (2013)
Viatmin B1	381–1151 μg/100g (DP)	Rózsa et al. (2019)
Viatmin B2	3183–5616 μg/100g (DP)	Rózsa et al. (2019)
Viatmin B9	291–671 μg/100g (DP)	Rózsa et al. (2019)
Viatmin B12	463–906 μg/100g (DP)	Rózsa et al. (2019)
Viatmin C	11.00–21.67 mg/100g (DP)	Rózsa et al. (2019)
Vitamin D2	1.95–3.68 mg/100g (DP)	Vieira Junior et al. (2021)
Vitamin E	124.25 ± 31.30 μg/100 g (DP)	Carneiro et al. (2013)
Flavor 5′-nucleotides	5.51 ± 1.35 mg/g (MY)	Chang et al. (2001)
a: MY, Content in mycelia of AbM		
b: DP, dry powder of AbM		
c: FW, Fresh weight of AbM		
d: FE, AbM fruit body after hydroalcoholic extract		
e: *, of glucose equivalents		
f: nd, not detected.		

## 3 Flavor and taste compositions

Chen (1986) claimed that mannitol was the taste-active compounds in mushroom sugars and 7.94 g/100g can be detected in AbM by Carneiro et al. (2013). On the other hand, the soluble sugars such as glucose (83.59 %), fructose (1.04 %) contained in mushrooms contribute to the sweet taste (Carneiro et al., 2013; Liu et al., 2017). Umami or palatable taste in mushroom contributed by the synergistic effect of 5′-nucleotides (5.51 mg/g in AbM mycelia) with MSG-like components. And aspartic (0.50 mg/g) and glutamic acids (not detected in AbM) were monosodium glutamate-like (MSG-like) components, which gave the most typical mushroom taste in AbM (Chang et al., 2001). In addition, Yang et al. (2022) suggested umami amino acids and nucleotides potentially enhanced the salty intensity of NaCl. With the increasing demand of low-salt foods, the synergistic effect of umami and salty could satisfy the need of consumers who tend to acquire a low-salt diet. Thus, umami fungus including AbM could be potentially used as the ingredient for future functional food development.

## 4 Bioactivities of AbM

### 4.1 Immunoregulation and Anti-fatigue effect

**Fig. 2:**
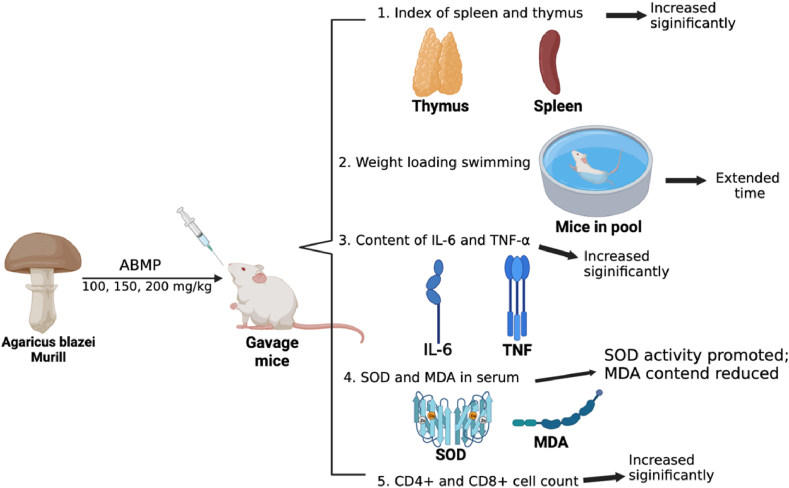
The experiment design of immunoregulation and antifatigue *in vivo* and test contents. The images were extracted from the BioRender app.

The spleen and thymus are essential central and peripheral immune organs of the body. As the largest secondary lymphoid organ in the body, the spleen could regulate T- and B-cell responses to these antigenic targets in the blood (Lewis et al., 2019). On the other hand, the cytokines such as tumor necrosis factor-α (TNF-α), Interleukin-6 (IL-6), and Interleukin-1β (IL-1β) were generated by immune cells, including macrophages and lymphocytes for the immunoregulation of human (Sun, 2017). Li and Wei (2017) indicated that the AbMP had significant bioactive effects on fatigue through above-mentioned indicators. Based on their research in Figure 2, the mice were divided into four groups, and the three groups received low, medium, and high gradient doses (100, 150, and 200 mg/kg BW per day, respectively) of AbMP. The other group was served the same amount of normal saline as the control group. After seven days of adaptive feeding, mouse thymus and spleen indices; serum IL-6, TNF-α; T lymphocytes: CD4^+^ and CD8^+^; were determined separately. Li and Wei (2017) demonstrated that AbMP significantly (*p*< 0.05) promoted the growth of the spleen and thymus in adult mice, and the thymus and spleen indexes of each dose group were increased to a certain extent compared with the control group. Furthermore, medium and high doses (150 and 200 mg/kg per day, respectively) of AbMP increased significantly (*p* < 0.05) the secretion of TNF-α and IL-6 to regulate the immune function of the body. Similarly, CD4^+^ and CD8^+^ as two vital T lymphocytes in adaptive immunity, and their number of cells significantly increased when treated with medium and high levels of AbMP. Thus, the authors declared that AbMP could significantly improve mice’s humoral, cellular, and non-specific immunity. Apart from that, superoxide dismutase (SOD) and malondialdehyde (MDA) are essential indexes to evaluate exercise fatigue (Li and Wei, 2017). SOD is the most important free radical scavenger in the body, and MDA is the final product of lipid peroxidation when free radicals act on lipids in living organisms, which reflects the degree of cell damage and can scavenge free radicals in the body (Peng et al., 2011). Thus, the authors also measured the concentration of SOD and MDA in mice after consumption of AbMP. The results exhibited that the concentration of SOD and MDA in the serum of the AbM polysaccharide dose groups of 150 and 200 mg/kg BW concentration was significantly different from that of the control group (*p* < 0.05). SOD is the leading free radical scavenger in the body, and the concentration of SOD in the serum of mice was significantly increased from 368.25 ± 24.23 to 478.62 ± 43.53 U/mg, which had significant antifatigue activity. In the experiment, the MDA concentrations were significantly lower than the control group (17.37 ± 2.46 and 23.54 ± 1.47 μmol/mg, respectively), which indicated that AbM extracts could reduce the process of free radical oxidation and resist the fatigue of the body.

**Fig. 3:**
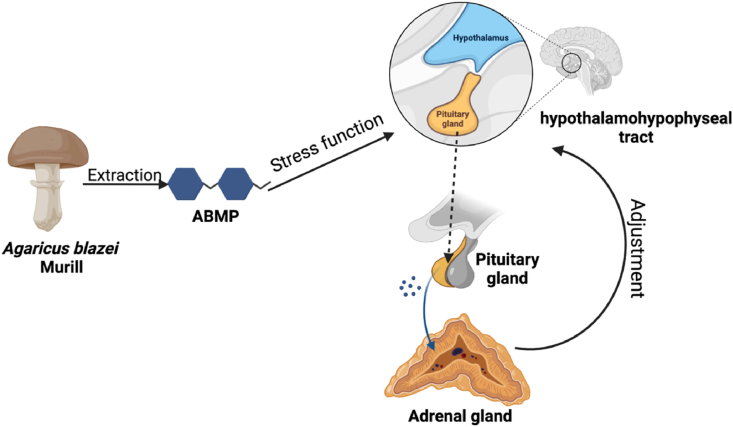
The AbMP stress function promotion diagram in HPA-Axis. The images were extracted from the BioRender app.

Additionally, peripheral fatigue is caused by the depletion of blood sugar and tissue glycogen due to the accumulation of lactic acid and ammonia metagarite. Glycolysis is the primary energy metabolism pathway during intense exercise, while blood lactate and hematuria nitrogen are the primary metabolites that indicate physical fatigue (Li et al., 2008; Li and Wei, 2017). Duan et al. (2003) found that the content of crude polysaccharides in the fermentation mycelium of AbM was much higher than that in the body. AbEXP1-a was a water-soluble polysaccharide which was isolated and purified by Li (2020) from crude extracellular AbMP, suggesting its antifatigue activity *in vivo* test. Fifty 35 g mice ingested 0.2 mL/kg BW of AbM polysaccharide per day, and their antifatigue indicators were measured after three weeks. The hematuria nitrogen and blood lactate levels after quantitative exercises in the test group were 7.84 ± 1.45 and 4.67 ± 0.99 mmoL/mL, respectively, which were lower than those in the control group (8.51 ± 1.22 and 7.66 ± 1.06 mmoL/mL); while the test group’s total swimming time was significantly prolonged to 47.95 ± 7.16 min comparing with the control group (39.42 ± 5.11 min). The previous results illustrated that the AbEXP1-a, an intracellular polysaccharide of AbM, had a powerful antifatigue effect. Additionally, the molecular weight of AbEXP1-a was within the range of macromolecular soluble active polysaccharides, which indicated that AbMP could effectively improve immunity. Chen et al. (2001) supplemented that AbM can produce a strong stress effect on the circulatory system in Figure 3. AbMP can improve the body's ability to use stress factors, then it can effectively increase the reflexes of the "hypothalamic-pituitary-adrenal cortex (HPA)" system in HPA-Axis and the body can quickly adapt to the harsh environment through continuous self-regulation (Duan et al., 2003). Therefore, they inferred that antifatigue effects of AbMP may correlate with the adjustment of HPA system.

Previous experiments have revealed the antifatigue and immunoregulative function of AbMP *in vivo* test and raised the underlying mechanisms by HPA-Axis system. However, AbMP’s absorption and metabolic efficiency in the human body still lack more clinical trials and further discovery is in demand. Besides, promoting the deep fermentation of AbM mycelium technology may benefit to developing functional foods due to its high production efficiency of crude polysaccharides.

### 4.2 Antidiabetic and anti-hyperglycemic effects

Type 2 diabetes is a group of complex heterogeneous metabolic diseases affecting a large population in worldwide. Current theories for the causes type 2 diabetes were attributed to the insulin-mediated deficiency of glucose uptake in muscle; impaired insulin action in the liver and disruption of adipocyte secretion, etc. *α* -Glucosidase is an enzyme that plays a crucial part in the final step of the digestion process, breaking down complex carbohydrates such as starch and glycogen into monomers. Meanwhile, it is worth noting that α -glucosidase could significantly increase postprandial glucose in patients with type 2 diabetes, and the abnormal value of the α-glucosidase enzyme could also lead to organ or tissue dysfunction, which in turn causes the development of type 2 diabetes (Lin and Sun, 2009; Wei et al., 2019). However, long-term consumption of existing antidiabetic drugs is supposed to occur multiple undesirable problems, for instance, side effects of insulin and oral hypoglycemic drugs (Etxeberria et al., 2012; Kim et al., 2005). Thus, Teng et al. (2017) clarified that there is a growing demand for natural products which can potentially inhibit α-glucosidase because of its role as the target of various drugs and inhibitors that regulate glucose metabolism in the human body. Fortunately, edible mushrooms may potentially prevent the secretion of α-glucosidase (Su et al., 2013; Stojkovic et al., 2019). Wei et al. (2019) demonstrated AbM extracts’ inhibition of α-glucosidase activity through the method designed by Chen and Kang (2014). 10–200 μg/mL AbM ethanol extracts (EE) and ethyl acetate extracts (EA) were placed in the 96-well microplate, followed by adding 0.1 U/mL α-glucosidase in sodium phosphate buffer. And then the absorbance value at 405 nm was measured by ultraviolet spectrometry, and the inhibition rate was calculated. It can be observed that the AbM EE and EA at 8 mg/mL exhibited 64.86% and 73.45% α-glucosidase inhibition capacity *in vitro.* Therefore, it can be hypothesized that AbM exhibited inhibition of the α-glucosidase under the action of organic extracts like EE and EA, which also offered potential use for anti-diabetic drugs. But Wei et al. (2019) findings still lacking data from *in vivo* and clinical trials and warrant further investigation, such as the inhibitory effects can be achieved *in vivo*, and whether the concentrations of the EE and EA extracts can be replicated in humans. And more clinical data may give noteworthy assistance to medical practitioners in the development of novel anti-diabetic drugs.

In addition, hyperglycemia may affect people with diabetes, which would induce severe complications that require emergency care, such as a diabetic coma ("Hyperglycemia in diabetes - Symptoms and causes", 2022). Kim et al. (2005) applied chromatography with Sephadex G-50 column to purify the AbM extracted *β*-glucans. And oligosaccharides (AO) were derived from hydrolyzing *β*-glucans with an endo-*β*-(1 → 6)-glucanase from *Bacillus megaterium.* The body weight and anti-hyperglycemia effects of β-glucans and AO were determined in diabetes rats, respectively. The diabetes rats were induced by 55 mg/kg streptozotocin through intraperitoneal administration for six weeks *in vivo*. The rats were divided into four groups; normal control; diabetic control; *β*-glucans treated, and AO treated. The body weight of rats in each group decreased compared with the control group, which met the agreement of Prince et al. (1998) research. The final body weight of rats treated with β-glucans and AO significantly decreased from 268.8 g to 252.6 g and 263.2 g, respectively (*p* < 0.05). Moreover, anti-hyperlipemic indicators: serum triglyceride and serum total cholesterol prominently decreased in *β*-glucans, and AO-treated groups compared with the control group (*p* < 0.05). Moreover, the hyperglycemia may also be inhibited by AbMP, as evidenced by Duan and Ma (2005). An alloxan-induced mice diabetes model was established, and concentrations of 4% and 8% AbMP were treated; the blood sugar content of mice with both AbMP concentrations in the experimental group was significantly lower than that of the diabetes model (*p* < 0.05). Thus, they claimed that a specific concentration of AbM polysaccharide had a hypoglycemic effect. On the other hand, Kim et al. (2005) studies also indicated that *β*-glucans and AO influenced insulin secretion; both *β*-glucans and AO treated rats’ insulin concentration in pancreatic islets elevated to 3.79 ng/mL and 4.89 ng/mL from 0.21 ng/mL in the diabetic control group.

AbM exhibited α-glucosidase suppression activity *in vitro*, effectively inhibiting type 2 diabetes. Additionally, the hyperglycemia in those with diabetes could be verified by certain concentration of AbMP, *β*-glucans and its derivation, AO may have glucose-lowering activity by stimulating insulin secretion in the pancreatic islets *in viv*o, which may be an essential source of latent functional food applications. However, the antidiabetic activity and effectiveness of AO in developing alternative drugs were twice superior to that of *β*-glucans (Kim et al. 2005). It can be speculated that the monosaccharide hydrolyzed from glucans may have a better antidiabetic effect than *β*-glucans and correlated anti-diabetes activities tests for different animal models and clinical trials should be conducted. In addition, during the production of AbM-based functional foods, suitable ratio of monosaccharides to *β*-glucans content may be more effective than aqueous solutions or AbMP extracts, but more *in vivo* and *in vitro* experiments need to be verified. Moreover, the underlying hypoglycemia mechanisms of cell models is quite limited, which still need further experiments.

### 4.3 Hepatoprotective activity

Liver diseases have become one of the leading causes of mortality worldwide in recent years (Dinakar et al., 2010). Traditionally, allopathic medical practice has no satisfactory for severe liver disease because the absent of reliable hepatoprotective drugs (Singh et al., 2003). Although several potential drugs have been identified, herbal as natural medicines were considered effective and safe alternatives to treat liver diseases, considering the side effects of modern medicines, doubtful efficacy, and safety. Thus, AbM as an edible medical fungus aroused researchers’ interest for its potential hepatoprotective activity (Madhu Kiran et al., 2012; Muthulingam et al., 2016). In general, elevated levels of serum marker enzymes are generally considered to be one of the most sensitive indicators of liver injury (Kapil et al., 1995). Increasing levels of alanine aminotransferase (ALT) and aspartate aminotransferase (AST) are indicators of cellular leakage and loss of functional integrity of cell membranes in the liver (Drotman and Lawhorn, 1978). Furthermore, alkaline phosphatase (ALP), the membrane-bound enzyme, is the prototype of mentioned enzymes that reflects pathological changes in bile flow (Ploa and Hewitt, 1989).

**Fig. 4:**
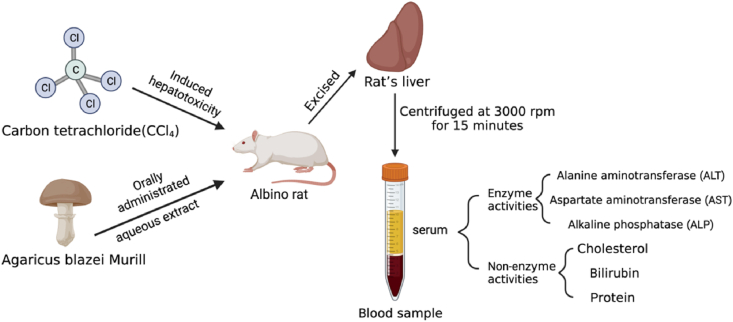
The experimental process of CCl4-induced liver injury with corresponding target testing items. The images were extracted from the BioRender app.

Muthulingam et al. (2016) applied the carbon tetrachloride (CCl_4_) model to discovered hepatic damage recovery by AbM extracts as illustrated in Figure 4. Mice were orally administrated with 250 and 500 mg/kg BW AbM aqueous extracts, and 1 ml/kg BW CCl_4_ was given at the same time for seven days. Initially, the activities of ALT, AST and ALP significantly improved in mice’s livers combating the CCl_4_ induced damage. However, serum elevated AST, ALT, ALP, bilirubin, and cholesterol activities significantly decreased, with an increase of serum protein levels after 28 days of AbM extracts continuous treatment (*p* < 0.05). Serum AST and ALT levels were reduced to normal levels, indicating the plasma membrane stabilization, which was consistent with the accepted view that serum transaminase levels return to normal with liver parenchymal healing and liver cell regeneration (Thabrew et al., 1987). Therefore, they claimed that AbM aqueous extracts can potentially inhibit the increase of ALP activity while reducing the elevated bilirubin level, suggesting that it may stabilize mice's liver function during CCl_4_-induced liver injury. Furthermore, Zhang et al. (2016) recognized the protective effect of AbMP liquid fermentation medium on alcoholic liver injury in mice. They found that when the extracellular polysaccharide content of AbM was 3.58 ± 0.13 g/L, the levels of ALT and AST in the test group were significantly decreased (*p* < 0.05), suggesting that the AbMP fermentation solution also had protective effect on alcoholic liver injury in mice. In a word, the aqueous extract of AbM and AbMP was evidenced its hepato-therapeutic effect *in vivo*, and it may be an alternative to existing drugs for the liver, but the effectiveness on the human liver is still unclear, and toxicological testing also needs to be refined to determine if functional food applications are possible. However, as a natural edible mushroom, AbM undoubtedly has broad application prospects for replacing medicines or health supplements for fewer side effects and a lower price.

### 4.4 Antitumor activity

There is currently little direct evidence for its specific anticancer substances and mechanisms (Fujimiya et al., 1998). Ergosterol was first isolated through gel column chromatography and claimed to be an antitumor compound in the AbM lipid fraction (Takaku et al., 2001). Sarcoma 180 – bearing mice were orally administrated ergosterol at doses of 400 and 800 mg/kg for 20 days, and which showed prominently inhibition of tumor growth without side effects induced by other cancer chemotherapy drugs such as thymus and spleen weights decreased. Additionally, they intraperitoneally administrated 10, 50, 100 and 200 mg/kg BW ergosterol to the mice, and the results indicated that ergosterol suppression ratio could reach 20.6 %, 57.1 %, 65.8 % and 84.7 %, respectively. Therefore, ergosterol or its metabolites were speculated to might be involved in inhibiting the formation of tumor neovascularization. Further research may focus on the possible synergistic effect by other bioactive compounds in AbM and determine the underlying mechanism of ergosterol action at the cellular level. On the other hand, a polysaccharide-protein complex named antitumor organic substance Mie (ATOM) was cultivated from AbM mycelia by Ito et al. (1997) and they verified *in vivo*. The ATOM highly exhibited its antitumor activity efficiency on the model of Sarcoma 180 in mice which were implanted ATOM subcutaneously at 10 and 20 mg/kg/day BW and detected its ability against Ehrlich ascites carcinoma, Shionogi carcinoma 42 and Meth A fibrosarcoma at 50 and 100 mg/kg/day. Moreover, the ATOM exhibited no direct cytotoxic action on tumor cells *in vitro*; thus, the authors indicated that the tumor growth inhibition was attributed to immunological host-mediated mechanisms. But the experiments for this polysaccharide-protein complex still remains at the level of animal models, more experimental data from human beings would be valid for deeper applications such as adjunct to an antitumor drug or the supplement to a chemotherapy drug. Apart from that, Mizuno et al. (1990) demonstrated that several polysaccharides, including FI_0_-a-*β* (Mw about 50,000), FA-1-a-⍺ (Mw about 2,000,000), FA-a-*β* and FA-2-b-*β* extracted from AbM which revealed a noticeable antitumor ability. They applied ion-exchange chromatography and gel filtration to purify and then isolate the polysaccharides and evidenced their antitumor activity using Sarcoma 180/ICR-JCL mice for their cytotoxicity *in vivo*. The control group mice had a tumor size of about 26 cm^3^ at the third week, and after intraperitoneally 10 mg/kg FI_0_-a-β, FA-1-a-⍺, FA-a-β and FA-2-b-β injected treatment for ten days, the tumor size had decreased to 7.88 cm^3^, 1.80 cm^3^, 0.87 cm^3^, 1.23 cm^3^ with 70.51, 93.32, 96.77 and 95.44 inhibition ratio, respectively.

Currently, most known compounds with tumor-killing activity and their synthetic derivatives had insufficient tumor selectivity; chemotherapy regimens only responded to a few tumor diseases, such as hematopoietic malignancies. However, Fujimiya et al. (1998) claimed that AbM inhibitory effect can enhance the host’s immunity against tumor growth. The authors separated acid-treated fraction (ATF) from AbMP and performed antitumor experiments on BaLB/c mice. By nuclear magnetic resonance (NMR) analysis, the main components of ATF with the highest anticancer activity were (1 → 4)-⍺-D-glucan and its branch (1 → 6)-β. Then they set up a model with syngeneic MethA tumor cells subcutaneously injected; the right flank was named primary tumor, and the left was metastatic one. The distant primary tumor in the right blank could be observed that was infiltrated by natural killer cells *in vivo*. Electrophoresis and DNA fragment assay could also clarify that apoptotic processing was exhibited in tumor cells by ATF induced directly *in vitro*. Moreover, flow cytometry analysis also exhibited that ATF could increase the expression of the antigen Apo2.7 on the mitochondrial membrane of tumor cells, while it did not affect normal mice and IL-2-treated splenic monocytes, thus, which could be confirmed selective toxicity of ATF polysaccharide to tumor cells.

**Fig. 5:**
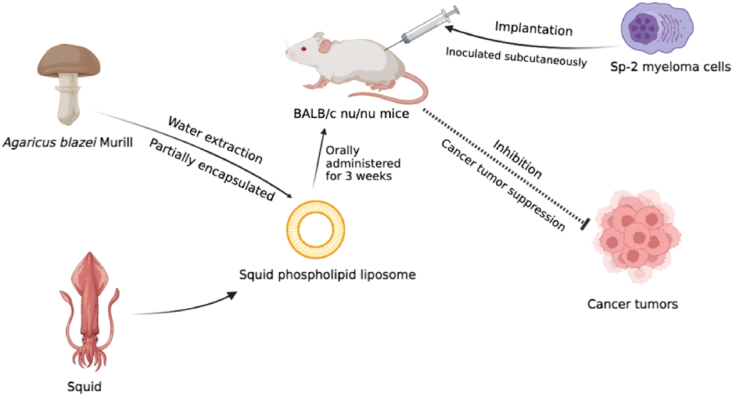
The squid phospholipid liposome combined with partially encapsulated AbM extracts for myeloma sp2 tumor suppression. The images were extracted from the BioRender app.

On the other hand, Murakawa et al. (2007) comprehensively found the AbM water extracts’ antitumor activity on myeloma cells with the combination of marine phospholipid liposomes. They designed experimental group as below; (1) control; (2) 1.0 mg/mL squid phospholipid liposome alone; (3) 0.5 mg/mL AbM water extract alone; (4) 1.0 mg/mL squid phospholipid liposome with 0.5 mg/mL AbM water extract in simple mixture; and (5) 1.0 mg/mL squid phospholipid liposome with 0.5 mg/mL AbM water extract partially encapsulated. The schematic diagram of group 5 is illustrated in Figure 5 below. The BALB/c nu/nu mice were inoculated subcutaneously with Sp-2 myeloma cells, and tumor sizes and weight were measured after 20 days of implantation. Previous studies illustrated that β-(1, 6)-glucan with β-(1, 3)-branched chains exhibited strong anticancer ability, which would have a promotion immunological competence when cooperated with liposomes (Kodama et al., 2002). The experiment results exhibited that β-glucan had a significant inhibition effect on the growth of myeloma sp2 tumor cells *in vivo* (*p* < 0.01), and the tumor disappeared in the partially encapsulated form administrated group (group 5). According to knowledge from Fujimiya et al. (1998) and Takaku et al. (2001), the mechanisms behind the suppression of the tumor cells may be attributed to AbM combined with liposomes cytotoxicity to tumor cells, interference with tumor angiogenesis. In addition, we could suggest that AbM constituents extract encapsulated with marine phospholipid liposomes could modulate tumorigenesis and might be useful for myeloma sp2 disease treatment based on Murakawa et al., (2007) report.

Generally, a few AbM extracts were demonstrated and identified for their antitumor activity in mentioned studies, but some unclear mechanisms remain partially understood. Polysaccharides such as FI_0_-a-β, FA-1-a-⍺, FA-a-β and FA-2-b-β, isolated from AbM aqueous which revealed antitumor activity and was responsible for immunological enhancement. On the other hand, (1 → 4)-⍺-D-glucan in ATF extracted from AbM had a selective antitumor activity that could induce apoptotic processing in tumor cells. Compared with other antitumor drugs, ATOM and ergosterol extracted from AbM both evidenced no cytotoxic effect on 180 sarcoma cells *in vitro*. More clinical trials may be conducted to verify its effectiveness on the human body, and then it could be used as a natural substitute for existing antitumor drugs. Moreover, the AbM water extracts encapsulated in marine phospholipid liposomes can regulate the occurrence of myeloma sp2 disease, which may be potentially helpful for treating myeloma disease in the future. In the future, more kinds of marine organisms not only the liposome binding of squid can be used to encapsulate AbM for antitumor experiments. These combinations may exist enormous commercial value for drug development and the emergence of novel functional food.

### 4.5 Anti-inflammatory effect

Inflammation is involved in the primary pathological process of many diseases, and which is an overreaction of the immune system. This may attribute to stimulation of xenobiotics, oxidative stress, or pathological processes such as injury and infection that are closely related to the occurrence, development, and cure of many diseases (Lawrence 2009; Zhao et al., 2004). Appropriate stimulation of macrophages by immune-related cytokines can protect the host from various pathologies and cancers, but excessive accumulation of pro-inflammatory cytokines and free radicals from macrophages can also lead to severe inflammatory diseases, such as asthma, inflammatory bowel disease (IBD) and rheumatoid arthritis (Medzhitov, 2008; Pahwa et al., 2022). In the past, anti-inflammatory drugs were broadly divided into two groups: steroids and non-steroids. In recent years, more and more attention has been paid to the anti-inflammatory effects of some plants and synthetic drugs with sound anti-inflammatory effects and mild adverse reactions (Zhao et al., 2004).

Zhao et al. (2004) suggested AbMP had anti-inflammatory effects on acute, subacute, and immune-epidemic inflammation *in vivo*. Thus, the authors established four mouse models: ear inflammation induced by paraxylene, foot swelling induced by carrageenan, cotton ball granuloma and adjuvant arthritis in rats. They injected them peritoneally with 40, 80 and 160 mg/kg AbMP to study their anti-inflammatory effects. As a type of rheumatoid arthritis, the primary lesion of adjuvant arthritis in rats was mainly a local acute inflammatory reaction. In their experiment, joint swelling could reach the peak at 18 h after inflammation and gradually decrease after three days. The results indicated that different doses of the AbMP group had a significant inhibitory effect on adjuvant arthritis in rats compared with the normal saline group after 18 h and three days based on the swelling rate of the inflammatory site (p < 0.05), and which had no significant difference with the indomethacin group. However, the mechanism of action remained unclear. Similar results could be obtained when acute inflammation in mice induced dimethyl benzene and subacute inflammation of tampon granuloma.

**Fig. 6:**
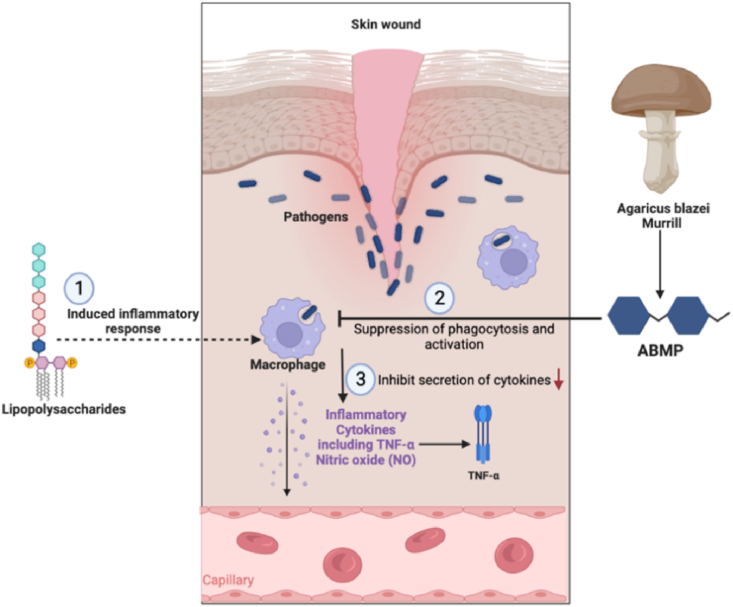
The potential mechanism of inhibition of inflammatory cytokines by AbMP. The images were extracted from the BioRender app.

As previously described, water-soluble AbMP (Mw 2.058 × 10^3^ ku) was extracted from AbM and purified using DEAE—sepharose fast flow chromatography column by Liu et al. (2017) and was generally acknowledged that have remarkable anti-inflammatory activities. As the main inflammatory factor, TNF-α can promote T cells to produce various inflammatory factors to promote inflammatory response, and nitric oxide (NO) would regulate macrophages to phagocytose pathogenic microorganisms and destroy pathogenic biological macromolecules. Although previous studies indicated that AbMP could stimulate the secretion of tumor necrosis factor TNF-α, IL-6, IL-10 and IFN-γ (Fu et al., 2015; Yang et al., 2013), while lipopolysaccharides (LPS) would over-stimulate macrophages when excessive (LPS) accumulated in the body and then release inflammatory mediators such as TNF-α, NO, and ILs, thereby triggering the body's inflammatory response (Zhao et al., 2010). In order to verify the anti-inflammatory activity and mechanism of AbMP, Liu et al. (2017) have studied the secretion of TNF and NO in lipopolysaccharide (LPS)-induced inflammation. TNF-α and NO secretion were measured when adding 62.5, 125, 250, 500 and 1000 μg/mL AbMP to 10 and 50 μg/mL lipopolysaccharide-induced inflammation mice model. The experimental results validated that AbMP significantly inhibited the secretion of these two cytokines, TNF-α and NO, in a concentration-dependent manner (p < 0.05), suggesting its anti-inflammatory activity. Specifically, the TNF-α secretion revealed a dose-response relationship at a relatively high concentration (250, 500 and 1000 μg/mL AbMP), and the NO concentration also significantly decreased when AbMP was lower than 250 μg/mL. However, the underlying mechanisms behind it were still unclear; the authors proposed it may be due to the suppression of phagocytosis and activation of macrophages by AbMP. As summarized below, AbMP extracted could potentially inhibit the production of inflammatory cytokines from macrophages which were induced by LPS (Figure 6). Another explanation would be the anti-inflammatory effects of down-regulated mRNA and inducible Nitric Oxide Synthase (iNOS) expression levels of TNF-α, but both hypotheses needed further addressed.

AbMP also exhibited anti-inflammatory activities in current clinical studies. AndoSan™ was confirmed as an anti-inflammatory medicine composed of AbMP, Hericium erinaceus (He) (14.7%) (LEE et al., 2000) and Grifola frondosa (Gf) (2.9%) (Adachi et al., 1994) can treat CD by reducing pro-inflammatory cytokines in patients (Therkelsen et al., 2016). Therkelsen et al. (2016) conducted a 21-day trial in 50 symptomatic CD patients randomly assigned to receive daily oral AndoSan™ or placebo. The AndoSan™ group had significantly improved scores for symptoms, fatigue, and health-related quality of life, while the placebo group had no significant changes (*p*<0.001). Therefore, they assessed that CD patients with mild to moderate symptoms might benefit from AndoSan™ as a safe supplement to conventional medication.

So far, AbMP has been demonstrated its potential medicinal value for various anti-inflammatory effects on acute, subacute, and immune-epidemic inflammation *in vivo,* and other diseases such as adjuvant arthritis, otitis, and foot swelling may exist in humans. The follow-up researchers should focus on establishing human inflammation models and exploring their anti-inflammatory effects on the body. Moreover, AbMP has exhibited its effectiveness in treating CD patients with mild or moderate symptoms. Natural alternative medicine that containing AbMP could be considered as a good choice for CD patients.

### 4.6 Antioxidant effect of AbM

It is well-known that the oxidation process is a fundamental reaction in living organisms while continuous production of reactive oxygen species (ROS) *in vivo* can lead to tissue damage and apoptosis. Human ageing and certain diseases, including diabetes, cancer and atherosclerosis, have been confirmed to be closely related to ROS (Calabrese et al., 2006; Eastwood, 1999; Halliwell and Gutteridge 1999; Morton et al., 2000). Thus, antioxidants aroused people’s interest in their potential organisms protected from ROS, such as superoxide anion radical (O_2_^•˗^) and hydrogen peroxide (H_2_O_2_). These by-products of normal metabolism are produced during respiration, hydrolysis, etc., and which can attack biomolecules such as lipids, proteins, DNA, and RNA, resulting in cellular or tissue damage associated with degenerative diseases (Jung et al., 1999). AbM is a natural food consisting of abundant polysaccharides, polyphenols and flavonoids that were considered an antioxidant with scavenging free radicals’ ability by scientists (Vellosa et al., 2006; Hakime-silva et al., 2013).

In recent studies, Wu et al. (2014) comprehensively evaluated the efficiency of extraction ways to obtain three natural polysaccharides (AbMP-F, AbMP-V, AbMP-A) antioxidant activity from AbM. AbMP-F, AbMP-V, and AbMP-A are defined as freeze drying, vacuum drying and air drying, respectively. The polysaccharides accounted for 12.28%, 11.54% and 11.19% of AbMP-F, AbMP-V and AbMP-A samples, respectively. Moreover, their uronic acid followed the same order for 28.19%, 25.72% and 24.87% for AbMP-F, AbMP-V and AbMP-A, respectively. The polysaccharides isolated from AbMP-F exhibited significant differences in their content (*p* < 0.05), suggesting freeze drying was a better way of AbMP extraction. The antioxidant activity was conducted for testing their radical scavenging ability, and four assays including hydroxyl radical, DPPH radical, ABTS free radical scavenging ability and Fe^2+^-chelating ability assay with ascending AbMP concentration. Ascending concentrations from 300, 600, to 900 μg/mL, all showed positive concentration-dependent to their corresponding antioxidant activities in all extracts for each assay. Specifically, at 300 μg/mL, all three types of AbMP exhibited more than 50% scavenging ability of ABTS free radical. Hydroxyl radical scavenging activity of both AbMP-F and AbMP-V exhibited relatively high levels at all concentrations, but the scavenging ability of AbMP-A to hydroxyl radicals was always lower than 50% in the concentration range of 0.1–1.0 mg/mL. But their chelating ability on Fe^2+^, scavenging activity on DPPH radicals, and two previous assays showed a decreasing order of AbMP-F>AbMP-V>AbMP-A. AbMP-F illustrated its higher antioxidant abilities on scavenging hydroxyl radical, DPPH free radical, ABTS free radicals, and ferrous ion chelating ability for its relatively highest polysaccharides yield and uronic acid content. The author also suggested that higher uronic acid content may potentially correlate with AbMP’s antioxidant activities. Hence, researchers could devote to discover the relationship of uronic acid with antioxidant ability. Additionally, the synergistic effect of uronic acid and AbMP alsoworth to be determined.

Hakime-silva et al. (2013) obtained AbM aqueous macerated extracts and they evaluated its ability against oxidative stress through various antioxidant assays including (1) inhibition of oxidative enzymatic process and cellular oxidative stress and (2) direct action over-reactive species. The oxidative enzymatic process of HRP and MPO was conducted by chemiluminescence with 100% suppression. Furthermore, the ability to scavenge ROS, HOCl and superoxide anion radical O_2_^•^^˗^ were evaluated and exhibited 62% and 87% inhibition rate *in vitro*, respectively. The authors also illustrated that AbM aqueous macerated extracts could reduce ROS levels by inhibiting various reactive species or interfering with enzymatic ROS generators. As for cellular oxidative stress, the oxidative burst of polymorphonuclear neutrophils (PMNs) was suppressed by 80% in their experiment. Another assessment of AbM antioxidant activities was evaluated by Wei et al. (2019). AbM ethanol extracts (EE) and ethyl acetate extracts (EA) were analyzed by DPPH, ABTS radical scavenging, reducing power using K_3_Fe(CN)_6_ *in vitro*. In the DPPH study, the EE and EA exhibited 54.91% and 56.01% scavenging ability at 500 μg/mL, with no significant difference (*p* > 0.05). Furthermore, EE and EA in the present study contained 23 and 36 mg/g of total phenolic and organic content from AbM extracts, respectively, which mainly contributed to their antioxidant ability (Carvajal et al., 2011). However, the ABTS assays suggested EA had better scavenging activity than EE with lower IC_50_. The concentration of EE to inhibit 50% of ABTS was 304 μg/ml; in contrast, 161 μg/ml of EA achieved the same ratio. As for the reducing power of K_3_Fe(CN)_6_, the results illustrated a concentrated-dependent in AbM extracts. The author applied gradient concentration (156, 313, 650, and 1,250 μg/ml EE and EA), and the reducing activity showed 0.24, 0.44, 0.94, and 1.45 for EE and 0.28, 0.50, 0.95, and 1.29 for EA, respectively.

AbM also exhibited tissue peroxidative damage protection and inhibition of abnormal antioxidant levels in CCl_4_-induced hepatotoxicity in male albino rats. According to the previous study by Muthulingam et al. (2016), CCl_4_ can be used to induce liver tissue damage in rats. The control experiment was adopted as follows: (1) control group (no treatment), (2) only AbM extracts treatment and (3) only CCl_4_ treatment, (4) AbM extracts then treated with CCl_4_ and (5) treatment with CCl_4_ followed by AbM extracts treatment. Various enzymes in serum, such as ALT, AST and non-enzyme antioxidants glutathione, vitamin C, and vitamin E were determined. Nonenzymatic antioxidants such as reduced glutathione (GSH), vitamin C and vitamin E played an excellent role in protecting cells from oxidative damage. GSH in the blood maintains cellular levels of the active forms of vitamin C and vitamin E by neutralizing free radicals (Aldridge, 1981; WINKLER, 1992). Compared with the control group, the nonenzymatic antioxidant levels in the experiment treated with AbM extracts were increased, while the percentage of vitamin C, vitamin E, and GSH in rats decreased from 100% (control group) to 65.7%, 46.8% and 60.2% in the CCl_4_ treated group after AbM extracts adding, respectively, which verified that the AbM extract was involved in inhibiting oxidative damage. In addition, when GSH levels were reduced, so did the levels of vitamin C in the cells, suggesting that GSH, vitamin C, and vitamin E are closely related to each other, which also indicated that the AbM extract got involved in the process of inhibiting oxidative damage (Al-Dbass, Al- Daihan & Bhat, 2012).

In conclusion, AbM is a natural source of antioxidant compounds such as polysaccharides and phenolic compounds, and all of them have shown decent antioxidant activities in *vivo* and in *vitro.* Table 2 summarizes the antioxidant constituents with their activity test in previous studies. Moreover, the freeze-drying method is a better way to prepare AbMP, which can extract more polysaccharides, neutral sugar, and uronic acid, which may achieve higher efficiency on antioxidant substances production in the food industry.

**Table 2:** The AbM extracts and antioxidant activities with corresponding assays.

Extract and compounds	Methods	Activities	References
Polysaccharides (AbMP-F, AbMP-V, AbMP-A)	DPPH and ABTS, Hydroxyl radicals scavenging; Fe2+-chelating ability	Antioxidant activities followed decreasing order of AbMP-F>AbMP-V>AbMP-A; ABTS: 300 μg/mL for more than 50% scavenging ability	Wu et al. (2014)
AbM aqueous macerated extracts	Enzymatic and cellular oxidative stress (HRP, MPO and PMNs) and direct action over ROS (HOCl and O2• ˗)	Enzymatic oxidative stress of HRP and MPO: 100% suppression; Cellular oxidative stress of PMNs: 80% inhibition; HOCl and superoxide anion radical O2• ˗ were inhibited with 62% and 87%	Hakime-silva et al. (2013)
AbM ethanol extract and ethyl acetate extracts	Reducing power (K3Fe(CN)6), Hydroxyl, DPPH and ABTS radicals scavenging *in vitro*	Reducing power of K3Fe(CN)6: concentrated-dependent inhibition for EE and EA; DPPH: 54.91% and 56.01% scavenging ability at 500 μg/mL EE and EA, respectively; ABTS: EE 304 μg/ml and EA 161 μg/ml for IC50	Wei et al. (2019)
AbM aqueous solution	Activities of non-enzume antioxidants: glutathione, vitamin C, and vitamin E	Percentage of vitamin C, vitamin E, and GSH in rats decreased from 100% (control group) to 65.7%, 46.8% and 60.2% in the CCl4 treated followed by AbM added group, respectively.	Muthulingam et al. (2016)

### 4.7 Other health-promoting functions of AbM

In addition to the above-mentioned health functional improvement of AbM, it also exhibited its antibacterial infection, antimutagenic and anticarcinogenic effects on previous studies.

Food contamination and spoilage may lead to bacterial and viral infections, thus, the development of food additives with antibacterial activity to prevent food contamination has become necessary in the food industry (Zhao et al., 2015). Bernardshaw et al. (2005) launched the first report on the anti-infective effect of AbM *in vivo*; 10 μL water extract of AbM had reflected its inhibitive effect of moderately virulent Streptococcus pneumoniae serotype 6B activity on orally administered NIH/OlaHsd mice. Although AbM extract lacked antibiotic effect against pneumococcus *in vitro*, serum cytokine MIP-2 and TNF-α levels were elevated in AbM extract-treated mice, suggesting that the protective effect of AbM extracts was due to the involvement of the self-immune system. The experiment served that AbM extracts may initiate an immune response in animals and humans by boosting patients’ immunity.

According to the latest WHO estimates, cancer cases rose to 18.1 million, and the death toll could reach 9.6 million. Therefore, it is imperative to adopt novel strategies to prevent from the cancer. Shen et al. (2001) have studied the anticancer effects of two polysaccharides (AbMP-Ⅱ-α and AbMP-Ⅱ-β) on human leukemia cells *in vitro*. The crude polysaccharides of AbM were separated into five components (I-V) by DEAE-cellulose ion-exchange chromatography. Then the Sephadex G-200 gel filtration column was used to isolate the components Ⅱ into AbMP-Ⅱ-α and AbMP-Ⅱ-β. 50, 100 and 200 μg/mL of AbMP-Ⅱ-α and 5.8, 29 and 145 μg/mL AbMP-Ⅱ-β were used to investigate the effect of AbMP on the proliferation of leukemia cells. AbMP-Ⅱ-α revealed a limit inhibition effect at low concentrations. However, AbMP-Ⅱ-β can inhibit cell proliferation in a dose-wise fashion. On the contrary, the growth cycle suppression of leukemia cells was significant by AbMP-Ⅱ-α. At 100 μg/mL, the inhibition rate could reach 77.8%-98.4%. The inhibition rate could reach 25.51% after 72 h at a low concentration of 0.16 μg/mL. Admittedly, the dosage and duration of AbMP-Ⅱ-β were positively correlated with its inhibition, reaching 100% after 48–72h treatment at a high dose of 20–100 μg/mL. Bertollo et al. (2022) comprehensively concluded that AbM had some therapeutic potential in terms of anti-cancer effects as well as prevention and as adjuvant therapy to chemotherapy and radiation therapy. The regulation of the immune system is the key to its anti-cancer effect. Immunomodulation of AbM interfered with the activity of cytochrome P450 enzymes to increase chemotherapeutic function (Hashimoto et al., 2002). In addition, the authors mentioned that the low molecular weight AbMP (LMW-AbMP) in mushrooms could inhibit the activity of TNF-α and other cytokines related to nuclear factor kappa B (NF-kB) activation, thereby reducing the stimulation of NF-kB and E-activation of selectins. At present, the mechanisms behind the anti-carcinogenic activity of AbM may not be investigated enough in previous findings which might need more clinical trials to support their findings.

On the other hand, several *in vitro* studies have indicated that AbM extracts were involved in the inhibition of mutagenicity of benzo (a) pyrene in the Ames Salmonella microsome assay (Osaki et al., 1994; Ito et al., 1984). Delmanto et al. (2001) decided to investigate the anti-mutagenic effect of AbM *in vivo* using the models of mouse bone marrow micronucleus test (MNPCE) and peripheral blood micronucleus test (MNRET). The results exhibited the antimutagenic effect of oral gavage of 0.025 g/mL AbM extracts on 25 or 50 mg/kg cyclophosphamide (CP) intraperitoneal induced mice. It is worth noting that no single AbM lineage was found to have anti-mutational activity in MNT tests. In other words, the single lineage did not reduce CP-induced micronucleus frequency in bone marrow or blood in mice compared to the mixed lineage. The authors speculated that the antimutagenic component was not evenly distributed across lineages and/or is not evenly present in mushrooms at different times of the year. Thus, further investigation should focus on specific bioactive compounds in AbM extracts that act on the antimutagenic effect. Apart from that, the determination of a single sequence with anti-mutation ability can be helpful for the subsequent development of drugs and functional foods.

## 5 Impact of food processing

AbM as a functional food, is commercially cultivated in many countries. However, few studies have mentioned about their impact in food processing. In fact, different extraction methods of AbM compositions would potentially affect its biochemical activity and lead to numerous extraction efficiencies during food commercial production. The content of crude polysaccharides in the fermented mycelium of AbM was much higher than AbM fruit body; thus, the research submerged fermentation technology may greatly benefit AbM in the future application. Moreover, both boiling and microwave treatment of AbM significantly reduced the content of glucose, galactose, vitamin C and mannose, but no significant changes in their lipids content. Boiling and microwaving significantly reduced the species and relative content of AbM phenolic compounds, resulting in reduced antioxidant capacity such as DPPH scavenging activity (Sun et al., 2011). These effects need to be taken into account when calculating food intake from cooked AbM. On the other hand, Wu et al. (2014) suggested freeze-drying is a relatively better way to extract AbMP with higher uronic acid content and antioxidant effect than vacuum drying and air drying. Therefore, selecting suitable extraction methods and parts plays a crucial role in the study of the biological activity of AbM and maintain the nutritional value of AbM as much as possible. Moreover, the impact during food processing of several cooking methods such as steaming, frying and grid splitting need further discoveries. At present, the related articles on AbM food processing are quite limited; there are few studies on the effects of different processing methods on the changes of nutrient components and biochemical activity functions, and more comprehensive studies on the effects of cooking methods on AbM can be conducted in the future.

### 5.1 Discussions and future application of AbM

**Fig. 7:**
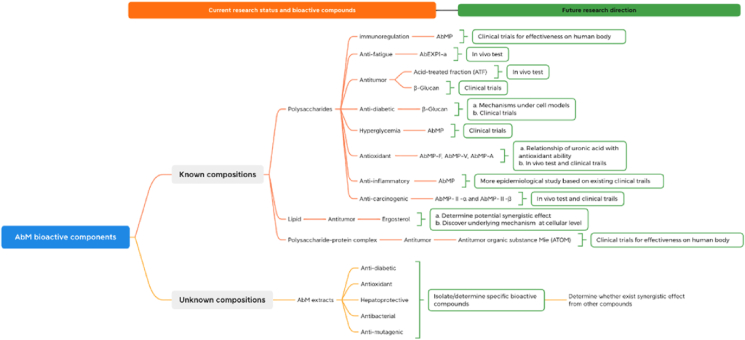
The bioactive constituents with their target functions and future research directions of AbM. The images were generated by XMind app.

AbM as a medicinal mushroom has showed several health-promoting effects *in vitro* and in *vivo*. Polysaccharides were the widest extracts isolated using heated aqueous or organic solvents such as ethanol and ethyl acetate. According to previous studies, AbMP was believed to affect its mechanism involved in the modulation of innate immunity. More specifically, AbM played a vital role in regulating macrophage function. AbMP could regulate cytokines secreted by macrophages, thereby reducing the production of inflammation. Besides, the prominent role of AbMP is to enhance and/or activate the macrophage immune response, resulting in immune regulation, wound healing and other therapeutic effects. These actions can be owned to the purified β-glucan in AbM that can act on various cellular receptors related to the initiation of immune responses. Nevertheless, there were no high-quality clinical trial data available to evaluate the actual clinical efficacy of purified *β*-glucan or compounds containing *β*-glucan (Chan et al., 2009). Well-designed clinical trials can be conducted to verify the effectiveness of β-glucans on human immunity and cancer cells in the future. In conclusion, AbMP as a natural plant polysaccharide which offers unique opportunities as a novel therapeutic adjuvant with beneficial immunomodulatory properties (Schepetkin and Quinn, 2006). Apart from that, AbM extracts can be prepared without extraction, exhibiting remarkable antidiabetic and antibacterial effects. Lipid and polysaccharide-protein complex mainly showed antitumor effects *in vivo*. These above-mentioned bioactive constituents with their target functions were concluded in Table 3. And their future research directions were also summarized in Figure 7.

**Table 3:** The main bioactive compounds found in the AbM, doses and administration routes, and target functions.

Bioactive substances	Components	Dose and/or administration route	Function	References
Polysaccharide	AbM polysaccharides (AbMP)	100, 150, and 200 mg/kg BW; oral	Immunoregulation	Li and Wei (2017)
Polysaccharide	AbEXP1-a	0.2 mL/kg BW; ingest	Anti-fatigue	LI (2020)
Polysaccharide	AbMP	100, 150, and 200 mg/kg BW; gavage	Anti-fatigue	Li and Wei (2017)
Polysaccharide	β-Glucans	2% β-Glucans; oral	Anti-diabetic	Kim et al. (2005)
Polysaccharide	β-Glucans	0.5 mg/mL AbM water extract; oral	Antitumor	Murakawa et al. (2007)
Polysaccharide	AbMP	4% and 8%	Hyperglycemia	Duan and Ma (2005)
Polysaccharide	AbMP (AbMP-F, AbMP-V, AbMP-A)	300, 600, and 900 μg/mL (*in vitro*)	Antioxidant	Wu et al. (2014)
Polysaccharide	Acid-treated fraction (ATF)	1 mg; injected intratumorally	Antitumor	Fujimiya et al. (1998)
Polysaccharide	AbM polysaccharides (AbMP)	40, 80 and 160 mg/kg;intraperitoneal injection	Anti-inflammatory	Zhao et al. (2004)
Polysaccharide	AbMP-Ⅱ-α and AbMP-Ⅱ-β	50, 100 and 200 μg/mL of AbMP-Ⅱ-α and 5.8, 29 and 145 μg/mL AbMP-Ⅱ-β (*in vitro*)	Anticarcinogenic	SHEN, SUN, LIU & GU (2001)
Polysaccharide	FI_0_-a-β, FA-1-a-⍺, FA-a-β and FA-2-b-β	10 mg/kg BW; Intraperitoneal injection	Antitumor	Mizuno et al. (1990)
Polysaccharide	AbMP (Mw 058 × 103 ku)	62.5, 125, 250, 500 and 1000 μg/mL (*in vitro*)	Anti-inflammatory	Liu et al. (2017)
Polysaccharide-protein complex	Antitumor organic substance Mie (ATOM)	10 and 20 mg/kg/day and 50 and 100 mg/kg/day; subcutaneous	Antitumor	Ito et al. (1997)
AbM extracts	–	8 mg/mL EE and EA (*in vitro*)	Antidiabetic	Wei et al. (2019)
AbM extracts	–	0.5 g/kg; oral	Antioxidant	Al-Dbass, Al- Daihan & Bhat, (2012)
AbM extracts	–	250 and 500 mg/kg BW; oral	Hepatoprotective	Muthulingam et al. (2016)
AbM extracts	–	10 μL AbM aqueous; oral	Antibacterial	Bernardshaw et al. (2005)
AbM extracts	–	0.025 g/mL; orally gavage	Antimutagenic	Delmanto et al. (2001)
AbM extracts	AbMP	3.58 ± 0.13 g/L;gavage	Hepatoprotective	ZHANG, LIN, DONG, WANG & HAN, (2016)
AbM extracts	–	100 μL AbM aqueous (*in vitro*)	Antioxidant	HAKIME-SILVA et al. (2013)
AbM extracts	–	156, 313, 650, and 1,250 μg/ml EE and EA (*in vitro*)	Antioxidant	Wei et al. (2019)
Lipid	Ergosterol	10, 50, 100 and 20 mg/kg BW; intraperitoneal 100, 200, 400 and 800 mg/kg BW; oral	Antitumor	Takaku et al. (2001)

According to Murakawa et al. (2007) research, encapsulating AbM with marine organisms can contribute to a better anti-tumor activity, thus, it can be speculated that the liposomes of the organisms may be able to stimulate the activity of AbMP. Whether the combination of AbM and liposomes can produce superior immunomodulatory function during drug treatment is worthy of more *in vivo* data to validate. In addition, AbM contains a variety of biologically active substances, and most experiments currently utilized its aqueous solution of AbM for experiments. On the other hand, many of the immunomodulatory substances may have synergistic effects on the target function. Therefore, the purification and separation of the specific substances in AbM could more effectively distinguish and identify biological activities and functions in subsequent studies. The bioactivities of AbM have been based mostly on *in vitro* and *in vivo* studies in mice. More clinical data and epidemiological investigations are needed to evaluate immunomodulation in humans at effective ingested doses of AbM as well as the chemical identification and quantification of certain compounds. Moreover, the content of heavy metals and the dose intake of mushroom alkaloids and their derivatives are accompanied by potential toxicity and carcinogenicity and that should be evaluated more comprehensively in AbM. Despite the challenges, there is no doubt that AbM has advantages of easy growth, low price, and rich nutrients which has great potential to be utilized in the market as functional food and drug substitutes in the future.

## 6 Conclusions

AbM is considered a medical mushroom for its immunological, antitumor and anti-inflammatory activity, owned to its various bioactive compounds including polysaccharides, lipids, and phenols. On the other hand, freeze-drying method was considered as the comparatively better way to acquire AbMP with higher antioxidant effect. Meanwhile, deep fermentation of AbM mycelium and use of AbMP-derived AO may potentially provide a better choice for functional foods such as diabetes. Up to now, research on the immune regulation, diabetes, and antitumor impact of AbM is gradually deepening, while polysaccharides such as β-Glucans and ATF would be subjected to more epidemiological investigations. Taken together, most of the experiments are currently concentrated in test tubes and animals, and there are relatively few universal clinical trials for the effectiveness of human body. However, it is believed that there would seek broad application prospects in the future as natural therapeutic alternatives based on AbM extracts due to their abundant nutritional value and health promotion activities.

## Funding

This study is jointly supported by two research grants R202016 and R202107 from 10.13039/501100001747BNU-HKBU United International College.

## CRediT authorship contribution statement

**Kaiyuan Huang:** Investigation, Data curation, Formal analysis, Software, Validation. **Baojun Xu:** Conceptualization, Methodology, Software, Supervision, Funding acquisition, Project administration, Validation, Writing – review & editing.

## Declaration of competing interest

The authors declare that they have no known competing financial interests or personal relationships that could have appeared to influence the work reported in this paper.

## Abbreviation

ABL.: Hemagglutinin
AbM: Agaricus blazei Murill
AbMP: Agaricus blazei Murill polysaccharides
AIDS: Acquired Immune Deficiency Syndrome
ALP.: Alkaline phosphatase
ALT.: Alanine aminotransferase
AST: Aspartate aminotransferase
ATF: Acid-treated fraction
ATOM: antitumor organic substance Mie
BW.: Body weight
CCl_4_: Carbon tetrachloride
CD: Crohn’s disease
CP.: Cyclophosphamide
DEAE: Diethyl-aminoethanol
EA: Acetate extracts
EA.: Ethyl acetate extracts
EE.: Ethanol extracts
EE.: Ethanol extracts
Gf: Grifola frondose
GSH.: Glutathione
He: Hericium erinaceus
HIV.: Human immunodeficiency virus
HRP.: Horseradish peroxidase
HRP.: Horseradish peroxidase
IL.: Interleukin
iNOS: Inducible Nitric Oxide Synthase
LPS.: Lipopolysaccharides
MDA.: Malondialdehyde
MNPCE: Mouse bone marrow micronucleus test
MNRET: Peripheral blood micronucleus test
MPO.: Myeloperoxidase
MS: Mass spectrometry
NF-kB: Nuclear factor kappa B
NMR.: Nuclear magnetic resonance
NO: Nitric oxide
PMNs: Polymorphonuclear neutrophils
ROS.: Reactive oxygen species
SOD.: Superoxide dismutase
TNF: Tumor necrosis factor
